# Supplementary material for: Mechanistic Insights from Transcriptomics: How the Glucose Transporter gltp1 Gene Knockout Enhances Monascus Pigment Biosynthesis in M. ruber CICC41233
Source: J Fungi (Basel). 2025 Dec 7;11(12):867. doi: 10.3390/jof11120867 (PMC12734223; doi:10.3390/jof11120867)
Supplement: Supplementary file 1 [file jof-11-00867-s001.zip › Figure S1. KEGG pathway enrichment of DEGs.pdf]

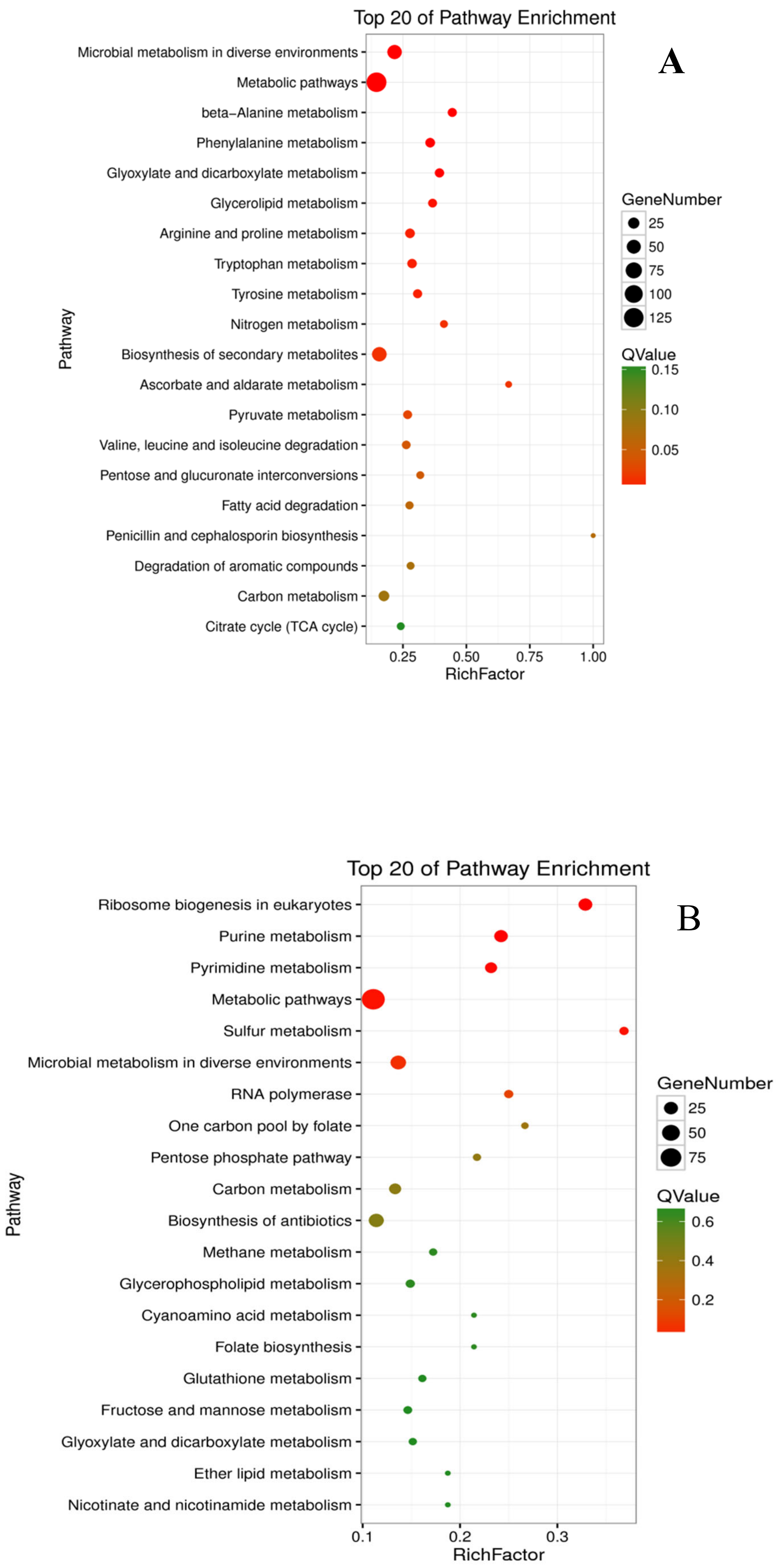
Top 20 of Pathway Enrichment
A
Microbial metabolism in diverse environments
Metabolic pathways
beta−Alanine metabolism
Phenylalanine metabolism
Glyoxylate and dicarboxylate metabolism
Glycerolipid metabolism
Arginine and proline metabolism
Tryptophan metabolism
Tyrosine metabolism
Nitrogen metabolism
Biosynthesis of secondary metabolites
Ascorbate and aldarate metabolism
Pyruvate metabolism
Valine, leucine and isoleucine degradation
Pentose and glucuronate interconversions
Fatty acid degradation
Penicillin and cephalosporin biosynthesis
Degradation of aromatic compounds
Carbon metabolism
Citrate cycle (TCA cycle)
Pathway
0.25
0.50
0.75
1.00
RichFactor
GeneNumber
25
50
75
100
125
QValue
0.15
0.10
0.05
Top 20 of Pathway Enrichment
B
Ribosome biogenesis in eukaryotes
Purine metabolism
Pyrimidine metabolism
Metabolic pathways
Sulfur metabolism
Microbial metabolism in diverse environments
RNA polymerase
One carbon pool by folate
Pentose phosphate pathway
Carbon metabolism
Biosynthesis of antibiotics
Methane metabolism
Glycerophospholipid metabolism
Cyanoamino acid metabolism
Folate biosynthesis
Glutathione metabolism
Fructose and mannose metabolism
Glyoxylate and dicarboxylate metabolism
Ether lipid metabolism
Nicotinate and nicotinamide metabolism
Pathway
0.1
0.2
0.3
RichFactor
GeneNumber
25
50
75
QValue
0.6
0.4
0.2

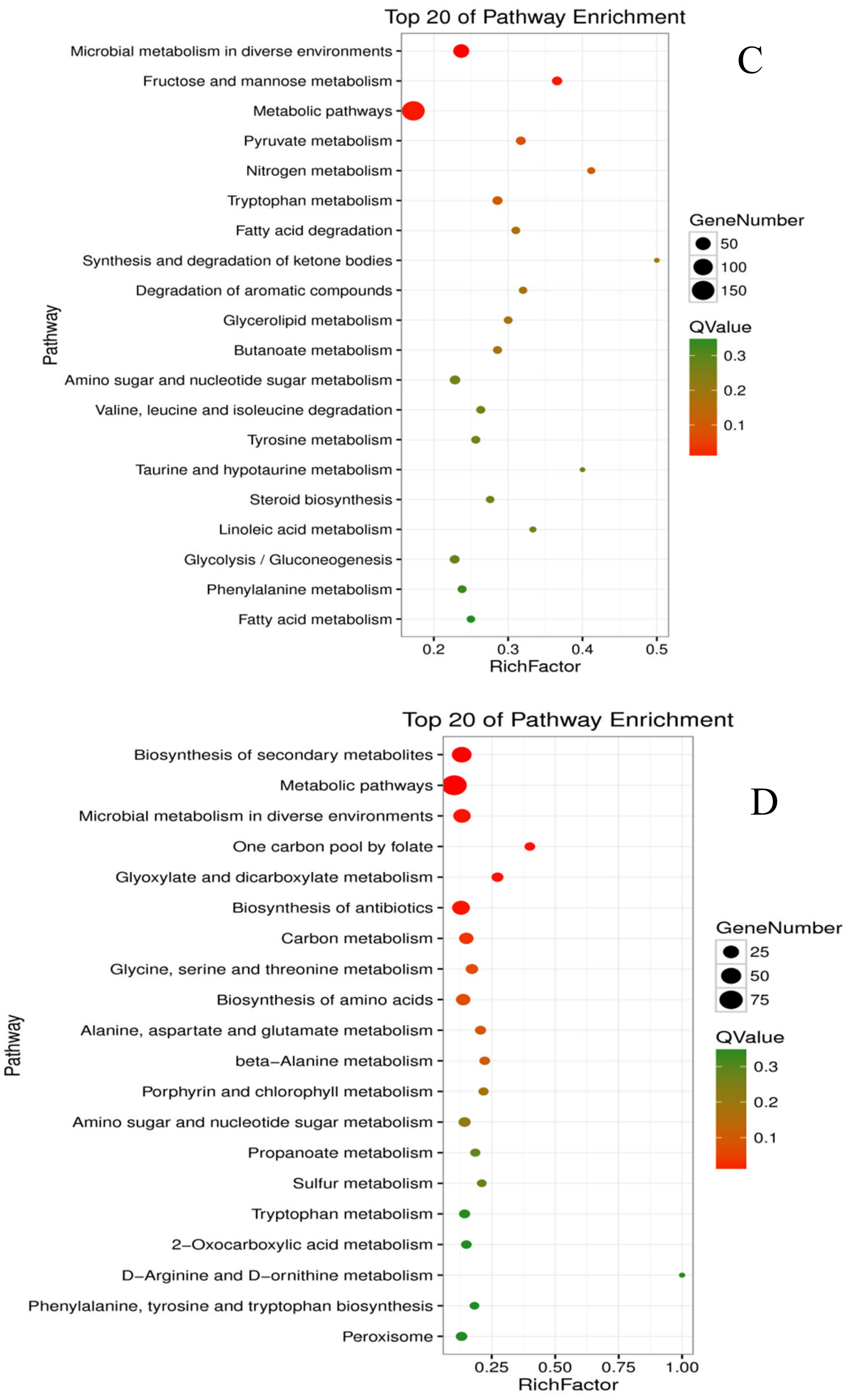

Figure S1. KEGG pathway enrichment of DEGs. (A) S01 vs. S02. (B) S03 vs. S04. (B) S01 vs. S03.(D) S02 vs. S04 (S01: 36 h sample for *M.ruber* CICC41233; S02: 36 h sample for *M.ruber* GLP24; S03: 144 h sample for *M.ruber* CICC41233; S04: 144 h sample for *M.ruber* GLP24)
